# Suicide Stigma among Medical Students in Puerto Rico

**DOI:** 10.3390/ijerph15071366

**Published:** 2018-06-29

**Authors:** Eliut Rivera-Segarra, Ernesto Rosario-Hernández, Paola Carminelli-Corretjer, Nelmit Tollinchi-Natali, Norka Polanco-Frontera

**Affiliations:** School of Behavioral and Brain Sciences, Ponce Health Sciences University, Ponce 00716, Puerto Rico; erosario@psm.edu (E.R.-H.); pcarminelli15@stu.psm.edu (P.C.-C.); ntollinchi15@stu.psm.edu (N.T.-N.); npolanco@psm.edu (N.P.-F.)

**Keywords:** stigma, suicide, medical students, Puerto Rico

## Abstract

Suicide is a global public health issue and the 10th leading cause of death, regardless of age, in the U.S. Puerto Ricans are U.S. citizens with one of the highest rates of suicide ideation and attempts (SIA) among all Latino sub-groups. Research has found that stigma is a risk factor for SIA. Medical students are an important group to target as they engage in routine clinical interactions with potential suicide victims, playing an important role in suicide prevention efforts. However, these efforts may be hampered by suicide stigma. The purpose of this study is to examine the correlates of suicide stigma in a sample of medical students in Puerto Rico. We implement an exploratory cross-sectional design using quantitative techniques. A total of 123 medical students participate in the study. Bivariate analyses suggest that gender is significantly correlated to suicide stigma (*p* < 0.05). Hierarchical regression analysis suggests that suicide literacy (β = −0.196, *p* < 0.05) and emotional reaction to suicide (β = 0.212, *p* < 0.05) predict suicide stigma. Although preliminary, these findings echo previous research regarding the importance of literacy and emotional reaction in the stigmatization process. Future research may develop intervention strategies aimed at reducing suicide stigma among medical students.

## 1. Introduction

Suicide is a global public health issue and the 10th leading cause of death in the United States (U.S.) [1]. It is estimated that about 45,000 people die as a consequence of suicide each year in the U.S. alone [1,2]. Latinos are one of the fastest growing ethnic minority groups in the U.S. and a particularly vulnerable population as they account for a large proportion of suicide deaths in the country [3,4]. Puerto Ricans are U.S. citizens living in the Caribbean archipelago who have one of the highest rates of suicide ideation and attempts (SIA) among all Latino subgroups, with estimates of 7.9% and 3.5%, respectively [5]. These patterns have increased in the aftermath of Hurricane María, with higher rates of completed suicide and SIA among Puerto Ricans [6,7]. As migration from Puerto Rico to the mainland U.S. increases, this becomes a major national public health concern. Thus, it is vital to address the social and cultural determinants that foster these health disparities among Puerto Ricans, such as the stigma of suicide.

Stigma is a social determinant of health that lead people to experience social rejection and poor health outcomes [8,9]. Recent research has suggested that suicide stigma is a risk factor for suicide attempts [10,11,12]. Stigmatization has also been recently identified as a potential barrier for suicide prevention treatment [13,14]. When people with past or current SIA experience suicide stigma it decreases their sense of self-worth, social support network and help-seeking behaviors [11,13]. Despite this, few research efforts address suicide stigma. Furthermore, most of these studies focus on the general population, neglecting to address fundamental actors in suicide prevention and treatment efforts such as physicians and medical students [15].

Physicians and medical students are an important group to target as they engage in routine clinical interactions with patients with potential SIA, playing an important role in suicide prevention efforts [16]. Research has documented that across all age groups, up to three quarters of people with SIA will interact with a health professional in a general practice setting before attempting suicide [17]. Furthermore, more than half of people who successfully commit suicide contact their physicians at least four weeks prior to their deaths [18]. However, recent research has documented that few physicians routinely engage in suicide risk prevention efforts during their general practice [19]. A recent study identified a similar pattern with medical students in the context of Puerto Rico, with only 32% of medical students engaging in direct suicide inquiry during simulated healthcare interactions as part of their training [20,21]. The literature has posited the possibility that this could be related to a lack of suicide literacy and a hardening of physicians’ and medical students’ attitudes towards patients with SIA at the end of their medical education [22]. In addition, some research suggests that negative emotional reactions could also play a role in avoiding inquiring about SIA in routine clinical interactions [19,23]. However, despite this, research has yet to address the relationship between these factors and suicide stigma. This is particularly troublesome as suicide stigma can worsen the ability to recognize SIA in others, which is a vital skill for suicide prevention efforts in clinical interactions [24]. Thus, the purpose of this study was to examine the correlates of stigmatizing attitudes towards suicide in a sample of medical students in Puerto Rico. We tested two specific hypotheses, which were the following: (1) suicide literacy will be inversely correlated to suicide stigma; and (2) emotional reactions will be positively correlated with suicide stigma.

## 2. Materials and Methods 

### 2.1. Study Design and Procedures

We implemented a cross-sectional exploratory study design using quantitative survey techniques. We invited a sample of first and third year medical students from two orientation seminars (e.g., residence interview process) to participate in our study at the beginning of their academic semesters and prior to gaining clinical rotation experience. Participants were selected by availability from one of the four accredited medical schools in Puerto Rico. This school was selected due to their ample pool of participants (approximately 100 new students annually). Potential participants contacted the researchers who then proceeded to explain in detail the objectives of the study. Participants met the following eligibility criteria: (1) be adults of legal age in Puerto Rico (21 years of age), (2) be a Spanish speaking person, and (3) be an active medical student. All medical students in Puerto Rico require at least a Bachelor’s degree in order to begin their medical education. This study was approved by the Ponce Health Sciences University Institutional Review Board to ensure the protection of participants involved (170306ER).

To estimate the sample size for the current study, we used the G*Power program [25]. In order to obtain a 95% power and a medium effect size (f^2^ = 0.15) with two predictor variables (suicide literacy and emotional reaction) and one control variable (gender). A total sample of 119 participants was estimated. However, a total sample of 123 medical students participated in the current study.

### 2.2. Measures

Socio-Demographic Questionnaire—This questionnaire, developed by the research team, addressed participant’s demographic information such as age, gender, income, marital status, among other variables of interest.

Literacy of Suicide Scale [26]—This was a Spanish translation of the 27-item original English language scale. This was a suicide ideation and attempt knowledge measure answered in a correct/incorrect/don’t know format. It has been used with medical students in other cultural contexts and it has shown acceptable reliability coefficients (α = 0.71). In this study, our Spanish translation also demonstrated good reliability coefficients (α = 0.83).

Emotional Reaction to Suicide Scale—This instrument was adapted and translated from the original Emotional Reactions to the Mentally Ill Scale [27] to reflect a suicide ideation case in Spanish language. This 9-item scale measured emotions such as pity, fear and anger and included a case vignette of a person with suicide ideation. Each item was rated in a 5-point Likert scale. In this study, our adapted and translated Spanish version had good reliability of 0.79.

Stigma of Suicide Scale Short Version—This was a translated and adapted Spanish version of the three-factor original English language short version of the scale to measure stigma towards people who successfully commit suicide [28]. This short version consisted of 16-items and had acceptable reliability with α = 0.70. Each item consisted of a one-word descriptor of a person who committed suicide, rated on a 5-point Likert scale from (1) strongly disagree to (5) strongly agree. In this study, our Spanish short version demonstrated a good reliability of 0.81.

### 2.3. Statistical Analysis

All analyses were conducted using IBM SPSS (v.21) (IBM, Armonk, NY, USA). We conducted descriptive statistics to determine the sample characteristics and we used bivariate and multivariate analyses to examine our study’s hypotheses. Bivariate analyses were used for identifying and confirming the control/covariates and multivariate analyses to test study’s hypotheses. All analyses significance was assessed at two-tailed *p* < 0.05. 

To manage the incomplete data from questionnaires, we used the “Expectation-Maximization” technique. This technique estimates the means and the variances of the part of the sample which satisfies the statistical criterion [29]. Kline highlighted the two steps of this technique. In the first step, the observations or incomplete data were imputed by predicted scores in a series of regressions in which each incomplete variable was retracted over the rest of the variables in a particular case. In the second step, the entire data set was estimated using the maximum likelihood method. These two steps were repeated until a stable solution was found. On the other hand, missing data from socio demographic data was not imputed because it was not used for the regression analysis. The only variable included (gender) was used as a control variable as it was found to be statistically significant in the bivariate analysis, and it did not have any missing value.

## 3. Results

### 3.1. Sample Characteristics

A total of 123 medical students participated in the study. A total of 52.0% of respondents were reported to be between 23 to 25 years of age. In terms of annual income, 57.7% of respondents reported an income of $40,001 or more. A total of 65.9% of respondents were in their first year of study. Respondents reported that only 34.2% knew a person who had attempted suicide and 17.9% who had committed suicide (see Table 1).

The percentage of females who participated was 56.1%, the percentage of males was 43.1% males, and the percentage of transgender people was 0.8%. This distribution is a typical one in research in Puerto Rico were there are statistically more women than men. We conducted bivariate analyses with all socio-demographic variables. In the case of gender, we compared stigma levels by gender via *t*-test. We found that on average, males had more stigma (M = 18.94, SD = 5.83), than females (M = 15.37, SD = 5.04). This difference was significant t(122) = 3.622, *p* = 0.001; moreover, a medium effect size was found, Cohen’s d = 0.66. For this reason, we included gender as a control variable in the regression analysis. 

### 3.2. Bivariate and Multivariate Results

To examine the relationship between the study’s main variables, we conducted a correlational analysis using Pearson product-moment correlation coefficient (see Table 2). Because gender was significant in the bivariate analysis, we included it as a control variable in the multiple regression analysis. Results showed significant correlations between gender, suicide literacy, and emotional reaction and suicide with suicide stigma. 

In addition, we conducted a hierarchical regression analysis with suicide stigma as a criterion variable. As results from the bivariate analyses suggested that gender was significantly correlated to suicide stigma, we used gender as a control variable in this analysis. On the first step, gender was significant (β = −0.189, *p* < 0.05); however, on the second step, only suicide literacy was significant (β = −0.214, *p* < 0.05). On the third step, emotional reaction to suicide was significant (β = 0.212, *p* < 0.05) and suicide literacy remained significant (β = −0.196, *p* < 0.05), while gender did not (β = −0.124, *p* = 0.162). These three predictors explained only 12.4% of the variance on suicide stigma (see Table 3). 

Following Cohen’s guidelines [30], we calculated the effect size (f^2^) of the regression analysis for predictor variables. This effect size gives an idea of the impact of the predictors on the criterion. In this way, on the first step when adding gender an f^2^ = 0.04 was obtained. When adding suicide literacy in the second step, effect size increased to f^2^ = 0.09, and when adding emotional reaction in the third step, effect size increased to f^2^ = 0.14. This effect size, obtained with the predictors, is considered a small size effect. However, it was close to the medium effect size’s threshold of f^2^ = 0.15. 

## 4. Discussion

In this study, we aimed to examine the correlates of suicide stigma among medical students in the context of Puerto Rico. The results confirmed both of the study’s hypotheses. Our findings identified suicide literacy and emotional reactions to suicide as predictors of suicide stigma in a sample of medical students in Puerto Rico.

Our tested model confirmed the study’s hypotheses. Our findings suggested that suicide literacy and emotional reactions both predicted suicide stigma among medical students in Puerto Rico. These findings echoed research findings from the mental illness stigma literature with medical students in other contexts regarding the important role of knowledge in the production and re-production of the stigmatization process [31,32]. Moreover, they also echoed findings from the suicide literature which emphasized the role of emotions (e.g., pity) in attitudes, particularly among survivors and family members [33,34]. However, it is important to note that these results should be interpreted with caution as these variables only explained 12% of the variance in this study. Thus, our results also suggested that there are other important variables that should be taken into account when addressing suicide stigma. For example, future studies could examine complex cultural values such as “familismo” (the idea that families are an important source of support) or “machismo” (traditional gender roles assigned to men) and how they relate to suicide stigma in Latino cultural contexts [35]. Furthermore, variables such as personal mental illness or previous suicide ideation or attempts need to be addressed, particularly among medical students who have an increasing prevalence of both mental illness and SIA [36]. 

Another interesting finding in this study was that men had the highest stigma levels in the sample. Although the results align with previous research findings regarding suicide stigma level differences by gender in other cultural and community contexts [27], gender did not seem to predict suicide stigma in this sample. Future studies should examine this further with bigger samples. 

Finally, another important finding was that only 34.2% of the sample knew someone with a history of suicide attempt. Thus, although not statistically significant, this finding seems to be constant with previous research on mental illness stigmatization, which suggests that personal contact with a person with a mental illness could reduce stigmatization [31,32]. In this sample the contact with a person with a suicide attempt or completion (i.e., friend, family member, neighbor) was limited which could explain the suicide stigma levels in the sample.

Some limitations of this study include the use of a convenience sample of medical students from only one institution, which is not representative of the majority of medical students in Puerto Rico. In addition, students who agreed to participate may have answered in a socially desirable way, which could potentially bias their answers and thus the study findings. Furthermore, the sample was relatively small, which also limits the generalizability of the findings. Finally, the sample consisted of students from the first and third years of medical school with limited clinical experience, which limits the study’s ability to infer how these results might be related to patient outcomes.

However, despite these limitations, this is one of the few efforts that aims to better understand suicide stigma correlates among medical students. However, there is still a need for research to address the role of other psychosocial variables and specific emotions in suicide stigma among this population. This is of vital importance in order to develop tailored suicide stigma reduction interventions. It is unlikely that medical students receive substantial training on suicide after completing medical school, which underscores the need for interventions to address suicide stigma at an early stage of their medical school training. It is important for medical schools to examine their curriculums and evaluate when and how the topic of suicide is covered. Because suicide is not solely related to a mental illness, it cannot be exclusively addressed when covering psychiatric training or rotations. There is a need to familiarize medical students with suicide beyond their exposure to psychiatric courses or rotations. In addition, there is a need for stigma research to document the link between the provider’s suicide stigma and specific patient outcomes. Although research documents the negative health consequences of stigmatization for patients (i.e., self-worth, health seeking behaviors, poor health outcomes), we know little about the impact on patient’s health when suicide stigma is experienced from a healthcare provider. Finally, future research may also want to explore the role of suicide stigma among medical students own mental health help-seeking behaviors as recent research has documented a high prevalence suicide ideation among medical students [36]. However, the role of suicide stigma in this process remains unknown. 

## 5. Conclusions

Suicide literacy and emotional reactions to suicide ideation seem to predict suicide stigma levels among medical students in the context of Puerto Rico. Future suicide stigma research and stigma reduction intervention efforts should consider addressing these psychosocial variables. 

## Figures and Tables

**Table 1:** Sociodemographic Information.

Variable	Frequency	Percent	*p*-Value
**Gender**			
Male	53	43.1	0.031 *
Female	69	56.1	
Transgender	1	0.8	
**Age (Years)**			
21–22	33	26.8	0.372
23–25	64	52.0	
≥26	26	21.1	
**Annual income**			
>$10,000	10	8.1	0.079
$10,001–$20,000	14	11.4	
$20,001–$30,000	10	8.1	
$30,001–$40,000	12	9.8	
≥$40,001	71	57.7	
**Year of study**			
First	81	65.9	0.257
Third	39	31.7	
**How many people do you know who have attempted suicide?**			
None	56	45.5	0.158
1–3	37	30.1	
3–5	5	4.1	
**How many people do you know who have died by suicide?**			
None	76	61.8	0.760
1–3	20	16.3	
3–5	2	1.6	

Note: *n* = 123; * Significant.

**Table 2:** Means, standard deviations, and correlations between variables.

	Variables	Mean	SD	1	2	3	4
1.	Gender			1			
2.	Suicide Literacy	16.02	4.38	0.18 *	1		
3.	Emotional Reaction—Suicide	22.47	5.52	−0.14	−0.11	1	
4.	Stigma	38.72	9.02	−0.19 *	−0.24 **	0.25 **	1

Note: *n* = 123; SD = Standard Deviation; * *p* < 0.05, ** *p* < 0.01.

**Table 3:** Hierarchical regression analysis having stigma as criterion.

Step	Predictor	B	Standard Error	β	R	R^2^	ΔR^2^
1	Constant	43.966	2.605		0.189 *	0.036	0.036 *
	Gender	−3.325	1.572	−0.189 *			
2	Constant	49.955	3.570		0.283 *	0.080	0.044 *
	Gender	−2.653	1.567	−0.151			
	Suicide Literacy	−0.440	0.183	−0.214 *			
3	Constant	40.814	5.123		0.352 *	0.124	0.044 *
	Gender	−2.177	1.548	−0.124			
	Suicide Literacy	−0.404	0.180	−0.196 *			
	Emotional Reaction—Suicide	0.347	0.142	0.212 *			

Note: *n* = 123; * *p* < 0.05.
